# Elective surgical referral guidelines - background educational material or essential shared decision making tool? A survey of GPs' in England

**DOI:** 10.1186/1471-2296-12-92

**Published:** 2011-08-30

**Authors:** Naomi Blundell, Sian Taylor-Phillips, David Spitzer, Steven Martin, Ian Forde, Aileen Clarke

**Affiliations:** 1 Freelance Researcher, Devon, UK; 2 Division of Health Sciences, Warwick Medical School University of Warwick, CV4 7AL, UK; 3 Barts & the London School of Medicine & Dentistry, Queen Mary University of London, E1 2AT, UK; 4 Department of Epidemiology and Public Health, University College, London, WC1E 6BT, UK

**Keywords:** Family Practice [MeSH], Primary Health Care [MeSH], Referral and Consultation [MeSH] Surgical Procedures, Operative [MeSH], Practice Guidelines [MeSH]

## Abstract

**Background:**

To investigate general practitioners' (GPs') attitudes to guidelines for elective surgical referral in England. To understand their use of guidelines, and attitudes to shared decision making in the referral decision.

**Methods:**

A questionnaire was developed which investigated attitudes to and use of guidelines. It was given to a stratified random sample 30% (n = 310) drawn from GP lists of 10 English health districts (primary care trusts (PCTs)). GPs were invited to respond online, by telephone, fax or post. Data were analysed using descriptive statistics and backwards stepwise logistic regression.

**Results:**

Responses were representative of GPs in England, but (despite up to 6 contacts per non-responder) the overall response rate was 41.6% (*n = *129; with the range across PCTs of 25-61%). Most responding GPs indicated support for referral guidelines but 18% reported that they had never used them. Less than three per cent reported use for most or all referral decisions. The odds of using guidelines decreased with increasing age, with a ten year increase in age associated with halving odds of use (OR = 0.53, 95%CI = 0.29-0.90). Over 50% of GPs wanted good access to electronic guidelines with expert information and advice on guideline availability. Almost all (>89%) GPs agreed with sharing referral decisions with patients. Female doctors (OR = 5.2, 95%CI: 1.02-26.3) were more likely to agree with this than male GPs as were those working in larger compared to small or single handed practices (OR = 5.3, 95%CI: 1.4-19.9).

**Conclusions:**

This group of responding GPs was supportive of guidelines but used them in different ways. Referral guidelines should have an educational component for background reading; include key messages for internalisation and application; and incorporate mechanisms to facilitate accessibility and appropriate shared decision making with patients.

## Background

The way general practitioners (GPs) in the UK are expected to provide care has changed considerably in recent years. Hundreds of national and local service frameworks and clinical guidelines have been distributed, in an attempt to improve the quality and evidence base for care and to minimise variation. At the same time, patients are encouraged to take a more active role in their own management, sharing key decisions with their practitioner to ensure that their care is tailored to their needs and preferences [1].

People are referred from primary to secondary care for a number of different reasons: including diagnosis, reassurance, further assessment of a known condition and often specifically for assessment for surgery [2,3]. However until recently, referral guidelines have not incorporated explicit reference to patient preferences, health status or quality of life [4].

One of the key determinants of demand in the NHS remains the GP's gate keeper role, whereby decisions about referral for further opinion, investigation or treatment are taken by GPs [5]. Access to secondary care is restricted to those whom the primary care practitioner consider appropriate for specialist care. However, there is substantial variation in referral rates in the UK [6]. There is a need to improve the appropriateness of referral for elective surgery and to introduce guidance so that both over- and under- referral are reduced [7].

Evidence suggests that GPs are more likely to use guidelines which are clear, concise, accessible, [8-10] evidence-based, from a credible source, [11-13] and relevant to everyday practice. However the evidence that guidelines alone are effective at changing clinician behaviour is limited [14-18]. Various models, building on the work of Rogers on diffusion of innovations and others [19] have been proposed both to explain why guidelines are not used as they should be and to suggest methods for improving the use of guidelines [20].

However, there are few studies which have investigated in detail the ways in which referral guidelines are used by GPs or the characteristics which affect their use of such guidelines particularly with regards to supporting or constraining patient choice.

Exploring these issues is timely. In England, health service reforms are proposed [21] which give GPs their own budgets in a climate of financial austerity, making them responsible for prioritising and commissioning secondary care. Reducing referral to secondary care for elective surgical intervention will be one of the key mechanisms available to GPs for reducing costs.

Primary care for many health care systems is organised similarly internationally [5] and findings are likely to be generalizable more broadly.

In this study, we aimed to use a survey to investigate two key research questions.

1. To what extent are referral guidelines used and what influences this?

2. Is shared decision making in the referral decision viewed as an important principle and what influences this?

## Methods

Questionnaire development: an 8-page self-completion questionnaire (Additional file 1: Appendix 1) was developed based on previous research [22] and the knowledge of the research team. An initial draft was tested with ten GPs, who were given a choice of how to provide feedback (either by commenting in writing or by taking part in cognitive "think-aloud" pilot interviews, in which they were asked to explain their thoughts as they worked through the questionnaire). Feedback from the pilot was used to revise questions and response options. The final draft of the questionnaire was pilot-tested with a further ten GPs to ensure clarity of language and question-wording, and to ensure that response options were exhaustive and mutually exclusive, where applicable.

Elective surgical referral guidelines were defined as "any structured paper or computer-based guide designed to assist those in primary care in making the decision whether or not to refer a patient to another professional where elective surgical intervention is one of the options for intervention." Factors considered likely to affect use of and attitudes to guidelines were; age, gender, experience and membership of professional bodies. Areas covered in the questionnaire are described below.

Areas covered in questionnaire:

• How and why GPs use guidelines - the role guidelines play in GPs' everyday practice. (This question was included as a result of pilot interviews).

• Support GPs believe they require for guideline use. (This question was included as a result of pilot interviews).

• Health conditions for which GPs have used, or currently use, referral guidelines, and conditions/topics where GPs believe new guidelines are needed. Response options for both topics consisted of an exhaustive list of common, non-urgent conditions amenable to elective surgical intervention. These were identified as follows. The Department of Health Hospital Episode Statistics (HES) data for England [23] were used to identify all conditions where a minimum of 10,000 elective procedures per annum were carried out, and where direct referral to a surgeon could be expected. Only conditions/topics which could be considered non-urgent at the time of surgery were included. The final list included: back pain, prostate problems, osteoarthritis of knee, stress incontinence, varicose veins, inguinal hernia, menorrhagia, cataract, sterilisation, haemorrhoids, osteoarthritis of hip, and infertility.

• Attitudes to patient involvement in decision making. Three existing alternative measures of GP attitudes to patient involvement in decision making were tested with ten GPs in the early stages of the questionnaire development process. Tools tested were the "Sharing" subscale of Krupat's two-part Patient-Practitioner Orientation Scale [24]; the patient involvement section of Ogden et al's four-part measure of GPs' and patients' beliefs about "patient-centredness" [25] and a tool developed by Elwyn and Edwards to measure the effect of an educational intervention on GP attitudes to shared decision making [26]. At the pilot stage, GPs were asked to comment on the acceptability of the content and language of each measure. Based on these comments, the Elwyn and Edwards tool [26] was selected as most suitable for inclusion in the final version of the questionnaire. In the version of this tool used for the survey,

• GPs were presented with a set of nine statements relating to their attitudes towards, and practice of, involving patients in decision making and sharing risk information. They were asked to indicate their level of agreement with each statement on a five point Likert scale, considering their approach to patient involvement in decisions regarding elective surgical referral.

• A "free text" question asking respondents for further comments they had about referral guidelines for elective surgery. Background data including demographic details; years since qualification; characteristics of practice; and membership of professional organisations.

Sampling: the sampling frame was all GPs in England. Since response rates can be low for GPs [27] responding to surveys, difficulties achieving a reasonable response rate were anticipated in planning and sample size calculation. After obtaining multi centre research ethics committee approval, (Scotland MREC; reference MREC/03/0/108), a sample of ten English primary care trusts was selected, broadly reflecting variation described in the Office of National Statistics' (ONS) data on classification of health areas (Table 1). The ONS categorises primary care trusts under eight headings or Supergroups which group together geographic areas according to key characteristics common to the population in that grouping using data on a range of factors including age group distribution, ethnic group distribution, household composition, housing, socio-economic information, employment and dominant industry sector [28]. We randomly selected 10 PCTs, one from each Supergroup with an additional one from each of three Supergroup including "Cities and services," "Coastal and Countryside" and "Prospering UK" to give a broader regional geographical coverage across England.

**Table 1:** Response by PCT

*PCT*	*ONS Supergroup*	*Geographical setting*	*Sample n*	*Response n*	*Response rate (%)*
A	Cities and Services	Outer London	23	9	39.1
B	Cities and Services	North West	16	4	25.0
C	Coastal & Countryside	South West	23	12	52.2
D	Coastal & Countryside	North East	34	20	58.8
E	London Centre	Inner London	32	9	28.1
F	London Cosmopolitan	Outer London	42	17	40.5
G	London Suburbs	Outer London	42	17	40.5
H	Mining and Manufacturing	North	28	12	31.6
I	Prospering UK	North West	47	14	29.8
J	Prospering UK	South East	23	14	60.9
Totals			310	129*	41.6

*One more than total as PCT could not be identified for one participant.

Research governance approval was obtained from each of the ten primary care trusts, and lists of practising GPs were requested and received from each. The GP lists of the primary care trusts were cleaned and validated using the NHS website available at the time [29] and by contacting practices by telephone where discrepancies were identified Lists were stratified into single handed and group GP practices. GPs had to be practising and registered on PCT lists. Locums/temporary workers were therefore excluded. A 30% random sample of GPs, stratified by practice size, was drawn from each primary care trust. The sample size was calculated to detect a 20% difference (90% versus 70%) in agreement to dichotomised outcome variables between two groups of GPs. This calculation used a significance level of 5%, at 80% power, assuming a 40% response rate.

Survey Methods: the survey was implemented in five stages. Anticipating non-response as a likely problem, measures were taken to enhance response rate at each stage. These are detailed below. Surveys were sent out between August 2005 and January 2006.

Staged implementation of survey to enhance response rate:

• Day 1: Eye-catching individually addressed postcard posted two weeks prior to initial mailing of the questionnaire. Postcard informed GPs about

■ study aims and methods

■ importance of involvement

■ full questionnaire arriving shortly

■ alternative methods for participation including online, telephone, fax, post

■ Prize draw incentive (for an iPod or a case of champagne)

• Day 14: Questionnaire posted to GPs, with Freepost envelope for return accompanied by an information letter which reminded GPs about alternative methods for participation

• Day 28: Non-responders sent a new copy of the questionnaire, accompanied by a similar letter, a Freepost envelope and an adapted version of the initial eye-catching postcard.

• Day 42 Non-responders sent a further personalised letter, notifying them that a member of the research team might contact them by telephone over the next few weeks and inviting them to take part in the survey by telephone interview. The letter reminded GPs of the various methods for participation (online, telephone, fax, post).

• Day 56-70 non-respondents in 5 PCTs were contacted 1-2 times by telephone and invited to take part by telephone, online, or fax.

Data handling: GPs choosing to respond using the web were given a unique entry number to complete their questionnaire. This system automatically completes their study database entry. Paper based responses and telephone responses were entered by study staff directly into the database. Data were cleaned. Analysis was undertaken using PASW statistics 17.0. Two dichotomous outcomes were defined as

i) GPs use or never use of referral guidelines and

ii) agreement (either 'strongly agree' or 'slightly agree') with the statement that "sharing decision making with patients is an important principle" versus neutral or "disagree" categories ('neither agree nor disagree', 'slightly disagree', or 'strongly disagree').

These were explored in backwards stepwise logistic regression analyses with age, gender, GP size, and personal list size as predictor variables. All four predictors were placed in the model, and assessed at each step against criteria to remain in the model (p < 0.1). The analysis stopped when all predictors remaining in the model met the criteria. Model evaluation, goodness of fit, and validation of predicted probabilities were calculated using the likelihood ratio test, the Hosmer-Lemeshow test and the c-statistic [30]. The Wald statistic was used to determine whether each independent variable was a significant contributor.

Descriptive statistics are also presented for why and how referral guidelines are used, the additional support required to help make the best use of referral guidelines, and issues with involving patients in referral decisions. Within this, the associations of participant characteristics with responses to three of the questions are presented according to a-priori hypotheses of effects. These hypotheses were i) that less experienced GPs are more likely to use guidelines because they are required to by someone else; ii) that less experienced GPs are more likely to state that they use guidelines when encountering difficult/unfamiliar circumstances; and iii) that younger respondents are more likely to want good access to electronic or internet based guidelines. For each hypothesis two-tailed 95% confidence intervals were calculated for differences between proportions using a version of Wilsons test [31].

## Results

Questionnaires were sent to 310 eligible GPs in ten PCTs in England after four GPs were excluded (1 retired, 1 moved to another practice, 2 locum GPs). Of the 310 eligible practitioners, 129 completed the questionnaire from 104 separate GP practices, giving a final response rate of 41.6%. Response rates varied by PCT from 25% to 61% and were lower in urban as compared to rural areas. (Table 1).

The majority of respondents were male and most were over the age of 45 years, with one quarter reporting that they had been fully qualified for less than ten years (*n *= 33, 25.8%,) and just over one in ten qualified for 30 or more years. More than three quarters of respondents were members of the British Medical Association (78.1%, *n *= 100) and about half were members of the Royal College of General Practitioners (43.8%, *n *= 56). Respondents were representative of the sample, and resembled GPs in England in terms of age, gender, practice size and membership of professional organisations Single-handed practitioners made up 4% (*n = *5) of respondents and 12.2% (*n *= 38) of the sample. (Table 2).

**Table 2:** Respondent characteristics

		*Respondents (n = 129)* *(%)*	*Sample* *(n = 310)* *(%)*	*England GPs* *(n = 34085)%^i^*
Age group				
	25 - 34 years	10.9	-	12.0
	35 - 44 years	28.9	-	34.1
	45 - 54 years	38.3	-	33.5
	55 - 64 years	18.8	-	18.0
	65 years or over	3.1	-	2.4
Sex				
	Male	61.2	62.4^ii^	59.6
	Female	38.8	37.6	40.4
Practice size				
	1	4.0	11.9	-
	2 - 3	34.1	36.6	-
	4 - 5	32.5	25.0	-
	6 - 7	7.1	8.4	-
	More than 7	22.2	18.1	-
				
BMA Membership		78	-	73^iii^
Royal College of General Practitioners Membership		43	-	43^iv^

i. DH General and Personal Medical Services Statistics, England and Wales, 30 September 2004 http://www.dh.gov.uk/en/Publicationsandstatistics/Statistics/StatisticalWorkAreas/Statisticalworkforce/index.htm accessed 30.03.2006

ii. Data on sex not available for 3 participating PCTs.

iii British Medical Association (personal communication)

iv *RCGP (Royal College of General Practitioners) (personal communication)

Twenty-three respondents (17.8%) stated that they had never used referral guidelines. Logistic regression analysis showed that although the effect of gender was not significant. (OR 3.4 95%CI = 0.9-12.7), the odds of using guidelines decreased with increasing age. A ten year increase in age was associated with halving of odds of use (OR = 0.53, 95%CI = 0.29-0.90). All respondents in the 25-34 year age group reported using referral guidelines, in comparison with 92% in the 35-44year age group, 81% in the 45-54 year age group and 58% in the 55-64 year age group.

The model was a good fit to the data (Hosmer and Lemeshow χ^2 ^(6) = 5.8, p = 0.45), and model predictions showed good agreement with actual outcomes (c-statistic = 0.749). (Table 3)

**Table 3:** Logistic regression analysis of whether respondents had ever used referral guidelines

Variable	Coefficient B	Standard Error of B	Wald Statistic	Degrees of freedom	**Sig**.	Odds Ratio Exp(B)	95% C.I. for EXP(B)	
							Lower	Upper
Gender (1 = male, 0 = female)	1.225	.673	3.315	1	.069	3.405	.910	12.730
Age Category	.658	.285	5.320	1	.021	1.930	1.104	3.374
Constant	-4.397	1.012	18.871	1	.000	.012		

a Variable(s) entered on step 1: gender, age, GP practice size, Personal list size. Likelihood ratio test χ^2 ^(2) = 12.689, p = 0.002, Cox and Snell R^2 ^= 0.10, Nagelkerke R^2 ^= 0.165, Hosmer and Lemeshow χ^2 ^(6) = 5.8, p = 0.45, c-statistic = 0.749

Responses indicated that guidelines were seen positively, and provided useful information for practitioner and patient. Just over a half (51.9%, *n *= 67) indicated that guidelines helped them to make good decisions, and nearly a third said that they helped them to explain or share information with patients "(30.2%, *n *= 39). Seventeen per cent said that they used guidelines (*n *= 22) because their local NHS organisation (PCT) required it. Only 13% of respondents cited being required to use guidelines by UK national bodies (e.g. National Institute for Health and Clinical Excellence (NICE), Department of Health) as a reason for using them. This differed by experience with 16% (13/79) of respondents with less than 20 years of experience as a GP and just 6% (3/49) of respondents with 20 or more years' experience cited this reason for using guidelines. However the difference was not significant. (Difference between proportions: 10% (95% CI -2% to 21%)). (Table 4).

**Table 4:** Number and percentage of GPs agreeing with questionnaire statements on why they used referral guidelines

	N*	*% of 129*
I believe they help me to make good decisions/improve quality of care	67	51.9
They help me to explain or share information about treatment decisions with patients	39	30.2
I am required to by my local hospital trust/local surgeons	31	24
I have never used referral guidelines	23	17.8
I am required to by my local PCT (e.g. as part of a "Choose & Book" scheme)	22	17
I believe they will reduce the possibility of litigation	19	14.7
I am required to by someone else (e.g. Department of Health, NICE, RCGP etc)	16	12.4
I use guidelines for another reason	8	6.2
The PCT offers incentives to encourage me to use them	2	1.6

*GPs were invited to tick more than one option

GPs indicated that the most important use of referral guidelines was in an educational capacity to provide help or information when their existing knowledge and experience did not provide the solution to a problem. 48.8% (*n *= 63) of GPs indicated, "I look at guidelines when I encounter difficult/unfamiliar circumstances." This response was more frequent amongst more recently qualified GPs, with 58.7% (44/75) of those who qualified less than twenty years ago selecting this option, compared to 40.4% (19/47) of those with 20 or more years of practice (difference = 18%, 95% CI = 0.1 - 35%).

The second most frequently cited use was again educational: reading a referral guideline "once or twice to improve knowledge about conditions" (41.9%, *n *= 54). Similarly 34.1% (*n *= 44) GPs indicated that they read guidelines once or twice and relied on memory in order to apply recommendations to individual patients. Only a very small percentage of GPs 2.3% (*n = *3) reported that they used guidelines on most or all occasions a referral decision was made. (Table 5).

**Table 5:** Number and percentage of GPs agreeing with questionnaire statements on how they used referral guidelines

	N*	*% of 129*
I look at guidelines when I encounter difficult/unfamiliar circumstances	63	48.8
I read guidelines once or twice for background education and/or to improve my knowledge of conditions	54	41.9
I read guidelines once or twice and rely on memory in order to apply recommendations to individual patients	44	34.1
I use guidelines in teaching	20	15.5
I use guidelines to help me audit my practice	12	9.3
I look at guidelines in most or all individual patient consultations where a referral might be necessary	3	2.3
Don't know	2	1.6
Use guidelines in another way	2	1.6

*GPs were invited to tick more than one option

GPs' were questioned about topics or conditions for which they had already used guidelines and also those conditions where they felt new guidelines might be beneficial. Three conditions were most commonly highlighted in both categories including lower urinary tract symptoms (prostate problems), infertility and back pain.

More than half of the responding GPs reported that they would like to have good access to electronic guidelines, a greater proportion of GPs aged under 45 years (66%, 33/50) expressed this preference compared to GPs aged 45 and older (44%, 33/75, difference = 22%, 95%CI: 4-38%). Information about available guidelines and their quality were also thought particularly important. In contrast, a relatively low proportion of GPs felt they would benefit from more training in how to use guidelines (8.5%, *n *= 11). (Table 6).

**Table 6:** Support identified by GPs' as helping them make best use of referral guidelines

	N*	*%*
Good access to electronic or internet based guidelines	66	51.2
Information telling me what guidelines are available	58	45.0
Expert advice on which are the best available guidelines	55	42.6
Regular updates telling me when new guidelines are produced	45	34.9
Good access to paper based guidelines	36	27.9
Technical support to help me find/access the best online/electronic guidelines	27	20.9
An internet source giving links to electronic guidelines	18	14.0
Technical support to help me USE online/electronic guidelines	16	12.4
General training on how to use guidelines	11	8.5
No support required - I choose not to use referral guidelines	9	7.0
No support required	5	3.9
Other type of support required	3	2.3

*GPs were invited to tick more than one option

Table 7 shows responses to questions about shared decision making in the referral decision. We grouped together the "agree" category responses. Nearly 90% of GPs agreed that sharing decision making with patients is an important principle (88.4%, *n = *114) Logistic regression analysis for respondents' views on the importance of the principle of sharing decision making with patients is shown in Table 8. The model was a good fit to the data (Hosmer and Lemeshow χ^2 ^(2) = 1.876, p = 0.39), and model predictions showed good agreement with actual outcomes (c-statistic = 0.723). Two variables remained in the model, gender and practice size. Female GPs (OR = 5.2, 95%CI = 1.02-26.3), and those from practices with more than 3 GPs (OR = 5.3, 95%CI = 1.4-19.9) were more likely to consider sharing decision making important. A large majority, 85.3% (*n = *110) said that they involved patients in referral decision making.

**Table 7:** Involving patients in the referral decision

Statement	Agree	Neither agree nor disagree	Disagree
I feel that sharing decision making with patients is an important principle	88.4	6.2	3.9
I feel "competent" in involving patients in decision making	79.1	12.4	5.5
I frequently involve patients in decision making	75.3	7	6.2
I have found that patients respond positively to involvement in decision making	74.4	15.5	8.6
Lack of time is a major problem in discussing treatment decisions with patients	58.9	24	14.9
I feel confident in discussing risk information about treatments with patients	55.8	23.3	15.5
Lack of available data** is a major problem in trying to share decisions	**47.3**	**34.9**	15.5
Many of my patients expect specific information to be provided in discussions about treatments	44.2	32.6	20.9
I feel my role is to direct patients rather than discuss risk information about treatments	17.1	22.5	55.8

** Available data" refers to local or national information on referral processes or evidence for benefits of referral

**Table 8:** Backwards stepwise logistic regression analysis whether sharing decision making with patients is considered an important principle

Variable	Coefficient B	Standard Error of B	Wald Statistic	Degrees of freedom	**Sig**.	Odds Ratio Exp(B)	95% C.I. for EXP(B)	
							Lower	Upper
Gender (1 = male, 0 = female)	1.643	.830	3.916	1	.048	5.171	1.016	26.322
GP practice size (0 = 3 or less, 1 = 4 or more)	1.673	.672	6.189	1	.013	5.327	1.426	19.898
Constant	-4.222	.917	21.184	1	.000	.015		

a. Variable(s) entered on step 1: gender, age, GP size, Personal list size.

Likelihood ratio test χ^2 ^(2) = 9.65, p = 0.008, Cox and Snell R^2 ^= 0.077, Nagelkerke R^2 ^= 0.162, Hosmer and Lemeshow χ^2 ^(2) = 1.876, p = 0.39, c-statistic = 0.723

Lack of time for involving patients was considered a problem by just under 60% of GPs while lack of available data was not so commonly regarded a problem.

In contrast to their overall support for the concept of shared decision making, and their own assessment of their *competence *in facilitating this, GPs were less inclined to feel *confident *in their skills in sharing decisions with patients. Whilst 81.6% said that they felt *competent *in sharing referral decisions, 59% felt *confident *in sharing such decisions.

## Discussion

We used a survey to investigate GPs' attitudes to and use of guidelines for elective surgical referral in England.

We anticipated a low response, and used evidence-based strategies to maximise response. Respondents were representative of GPs in England. Participating GPs indicated overall support for referral guidelines, but most appeared to use them in an educational capacity and very few (<3%) reported use on most or all of the occasions when a referral decision was made. Younger GPs were more likely to use guidelines than older male GPs. Over 50% of respondents wanted good access to electronic guidelines with expert information and advice on guideline availability. Topics identified for new guideline development included lower urinary tract symptoms, infertility and back pain. Almost all GPs (>89%) agreed with sharing referral decisions with patients. Female doctors were more likely to agree with this than male GPs as were those working in larger compared to small or single handed practices. This concurs with our findings on the incorporation of patients' preferences into referral guidelines [32].

A survey is arguably the most appropriate method for consulting a large sample of professionals across a wide geographical area. We ensured that rigorous and comprehensive processes of questionnaire design, piloting and administration were undertaken, but a brief scoping exercise exploring approaches to surveying GPs indicates that difficulties achieving a reasonable response rate should be expected [33]. Previous studies have demonstrated some measures which can help to improve response rates to varying extents, and such measures were rigorously employed in the present study including a prize draw for an iPod or a case of champagne [34].

Bearing in mind likely difficulties achieving a reasonable response rate, perhaps it is not surprising that a majority of our respondents was positive about the use of referral guidelines. They indicated that guidelines helped them make good decisions and/or improve the quality of care, in particular when confronted with a condition with which they were unfamiliar. They also indicated that they tended to use guidelines as background education.

With a response rate of just over 40 per cent, a reasonable assumption may be that many of the findings described above are only generalizable to a distinct group of GPs, perhaps those "compliers" who make greater use of guidelines than most. If this is the case, this paper potentially over-reports the interest of GPs in referral guidelines and under-reports "under-use" of guidelines. However, given these potential biases, it is of interest that just under one in five still felt able to report that they *never *used referral guidelines and only one in forty said that they used guidelines in most referral decisions where these were available.

Furthermore, as our respondents are potentially the very practitioners who, at present, make greatest use of guidelines, and actively seek out guidelines when they need them, they are likely to be the individuals with the experience and insight necessary to influence future guideline development decisions. Thus, while our findings cannot necessarily demonstrate the patterns of, and motivation for, guideline usage (or non-usage) amongst all GPs in England, they *can *inform and influence the format and content of new guidelines. This is particularly true in relation to the conditions for which new guidelines are needed, and the characteristics of a useful, user-friendly guideline.

We designed specific question in the survey e.g. on how and why guidelines are used de novo using background information, literature, interviews and piloting with GPs. This process should have ruled out uncertainties and ambiguities in questions, but we acknowledge that these questions have not been further tested for their validity and reliability. We did not include analysis of clustering effects in our data since the design effects due to clustering by GP practice was identified as low (e.g. 1.02 for use of referral guidelines).

Recent systematic reviews and studies of organisational behaviour are helpful in interpreting our findings. Our recent systematic review of guidelines for elective surgical referral [14] and a recent Cochrane review [35] indicate that whilst many interventions have been tried, the best method for enhancing use of evidence in referrals has still not been clarified. Evidence-based practice guidelines can have small effects although the uptake of evidence may be highly dependent on context [18]. Organisational studies indicate that the process of "uptake" of evidence might better be regarded as a process of "instrumentalisation" or internalisation [36]. This idea of internalisation fits with our findings of a lack of everyday use of elective surgical referral guidelines, with instead, use of guidelines as a background educational intervention or when difficult or unfamiliar situations occur.

## Conclusion

Our findings suggest that GPs consider referral guidelines valuable and would welcome an increase in access and availability of electronic guidelines for the referral decision. However we found that referral guidelines are not generally routinely used in relevant consultations. We therefore recommend redesigning referral guidelines so that they are more useful for the ways in which they appear to be actually used in practice.

- An educational component for background reading

- Key messages which can be internalised and applied in the consultation

- Clear indications of where and how shared decision making can be incorporated into referral consultations

- An accessible format to allow for easy location of information at the time of the consultation especially when a difficult or unfamiliar situation occurs.

## Ethics approval

A multi-centre research ethics committee approval was obtained for this research (Scotland MREC; reference MREC/03/0/108).

## Competing interests

The authors declare that they have no competing interests.

## Authors' contributions

NB, DS and AC carried out the survey. All authors participated in analysis and drafting of the manuscript and all authors read and approved the final manuscript.

## Pre-publication history

The pre-publication history for this paper can be accessed here:

http://www.biomedcentral.com/1471-2296/12/92/prepub

## Supplementary Material

Additional file 1 **Appendix 1**. Copy of GP Questionnaire. Click here for file
