# Supplementary material for: Elective surgical referral guidelines - background educational material or essential shared decision making tool? A survey of GPs' in England
Source: BMC Fam Pract. 2011 Aug 30;12:92. doi: 10.1186/1471-2296-12-92 (PMC3176475; doi:10.1186/1471-2296-12-92)
Supplement: Additional file 1 — Appendix 1. Copy of GP Questionnaire. [file 1471-2296-12-92-S1.DOC]

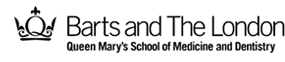
**The REFER Questionnaire**

**Survey of General Practitioners in England on the use of referral guidelines for non-urgent conditions**

**September 2005**

**Who is carrying out the survey?**

- The study is being carried out by the Academic Unit for General Practice and Primary Care in the centre for Health Sciences at Barts and the London School of Medicine & Dentistry , Queen Mary, University of London in collaboration with the Royal College of Surgeons for England and the London School of Hygiene and Tropical Medicine.

**What is the survey about?**

- This is a study about referral guidelines. We want your views on referral guidelines and how you use them.
- Referral guidelines are defined as “**any structured paper-based or computer-based guide designed to assist those in primary care in making the decision whether or not to refer a patient to another professional.”**
- We are particularly interested in your views on referral guidelines that are designed to assist GPs with the decision to refer adult patients to a surgeon for non-urgent conditions.

**Why is the survey being carried out?**

- The survey is being carried out as part of a 3 year study, the aim of which is to develop guidance to assist GPs in the process of referring patients to surgical specialties in secondary care; in particular providing GPs with support in the involvement of patients in the referral decision. The findings of this survey will inform and influence the way these guidelines are developed.

**What will happen to the results?**

- The findings of the survey will be used to inform the development process of referral guidelines which will begin in February 2006.

**What do I need to do?**

- We would like you to complete this short questionnaire, which should take only 5 - 10 minutes of your time. When you have completed the questionnaire please return it at your earliest convenience in the enclosed pre-paid envelope.
- Alternatively, if you would prefer to complete the questionnaire online, please visit www.ichs.qmul.ac.uk/research/gppc/publichealth and enter your unique reference number which you will find in the box below.
- If you would prefer to take part in the survey over the telephone please call our research team on 0207 123 4567.
- **All completed questionnaires will be treated confidentially and anonymously**. **Results will not be passed to anyone in a form that allows individuals to be identified** .

**How to contact us**

If you have any concerns or queries about this questionnaire please contact us on **0207 123 4567**

**Your questionnaire reference number:**

**123456**

# Your use of guidelines for the referral of adults to a surgeon for non-urgent conditions

## Have you ever used guidelines for the referral of adults to a surgeon for any of the following non-urgent conditions?

*(You may tick more than one option)*

| Back pain |  | 1 |
| --- | --- | --- |
| Osteoarthritis of knee |  | 2 |
| Varicose veins |  | 3 |
| Menorrhagia |  | 4 |
| Sterilisation |  | 5 |
| Osteoarthritis of hip |  | 6 |
| Prostate problems |  | 7 |
| Stress incontinence |  | 8 |
| Inguinal Hernia |  | 9 |
| Cataract |  | 10 |
| Haemorrhoids |  | 11 |
| Infertility |  | 12 |
| I have never used referral guidelines |  | 13 |
| Can’t remember |  | 14 |
| Other *Please write in box* |  | 15 |

## Thinking particularly of guidelines for the referral of adults to a surgeon for non-urgent conditions, which of the following options best describe how you use guidelines?

*(You may tick more than one option)*

| I look at guidelines in most or all individual patient consultations where a referral might be necessary |  | 1 |
| --- | --- | --- |
| I look at guidelines when I encounter difficult/unfamiliar circumstances |  | 2 |
| I never look at guidelines in individual patient consultations |  | 3 |
| I read guidelines once or twice and rely on memory in order to apply recommendations to individual patients |  | 4 |
| I read guidelines once or twice for background education AND/OR to improve my knowledge of conditions |  | 5 |
| I use guidelines to help me audit my practice |  | 6 |
| I use guidelines in teaching |  | 7 |
| Not applicable – I have never used referral guidelines |  | 8 |
| Don’t know |  | 9 |
| Other *Please write in box* |  | 10 |

## Thinking particularly of referral of adults to a surgeon for non-urgent conditions, which of the following options best describes why you use guidelines?

*(You may tick more than one option)*

| I use guidelines because **I believe they help me to make good decisions / improve quality of care** |  | 1 |
| --- | --- | --- |
| I use guidelines because **I am required to by my local hospital trust / local surgeons** |  | 2 |
| I use guidelines because **I am required to by my local PCT** (e.g. as part of a “Choose & Book” Scheme) |  | 3 |
| I use guidelines because **I am required to by someone else** (e.g. Department of Health) |  | 4 |
| I use guidelines because **the PCT offers incentives** to encourage me to use them |  | 5 |
| I use guidelines because **I believe** **they will reduce the possibility of litigation** |  | 6 |
| I use guidelines because **they to help me to explain or share information AND/OR treatment decisions with patients** |  | 7 |
| Not applicable – I have never used referral guidelines |  | 8 |
| Other *Please write in box* |  | 9 |

## Thinking particularly of referral to a surgeon for non-urgent conditions, do you think referral guidelines would be helpful for any of the following conditions?

*(You may tick more than one option)*

| Back pain |  | 1 |
| --- | --- | --- |
| Osteoarthritis of knee |  | 2 |
| Varicose veins |  | 3 |
| Menorrhagia |  | 4 |
| Sterilisation |  | 5 |
| Osteoarthritis of hip |  | 6 |
| Prostate problems |  | 7 |
| Stress incontinence |  | 8 |
| Inguinal Hernia |  | 9 |
| Cataract |  | 10 |
| Haemorrhoids |  | 11 |
| Infertility |  | 12 |
| None |  | 13 |
| Other *Please write in box* |  | 14 |

## Thinking particularly of referral of adults to a surgeon for non-urgent conditions, which of the following types of support would help you make best use of referral guidelines?

*(You may tick more than one option)*

| Information telling me **what guidelines are available** |  | 1 |
| --- | --- | --- |
| Expert advice on which are the **best available guidelines** |  | 2 |
| General training in how to use guidelines |  | 3 |
| Good access to **paper based** guidelines |  | 4 |
| Good access to electronic or internet **based** guidelines |  | 5 |
| Technical support to help me **find and/or access** the best online/electronic guidelines |  | 6 |
| Technical support to help me **use** online/electronic guidelines |  | 7 |
| An Internet source giving links to electronic guidelines |  | 8 |
| Regular updates telling me when new guidelines are produced |  | 9 |
| None of the above |  | 10 |
| Not applicable – I choose not to use referral guidelines |  | 11 |
| Other *Please write in box* |  | 12 |
|  |  |  |
|  |  |  |

# Involving patients in the referral decision

# Please indicate the extent to which you agree or disagree with each of the following statements.

|  |  | | **Strongly agree** | | | | **🡪** | | **Strongly disagree** | | | |
| --- | --- | --- | --- | --- | --- | --- | --- | --- | --- | --- | --- | --- |
|  |  | | **1** | | **2** | | **3** | | **4** | | **5** | |
| 1. I frequently involve patients in decision making | |  | |  | |  | |  | |  | |  |
| 1. I feel that sharing decision making with patients is an important principle | |  | |  | |  | |  | |  | |  |
| 1. I feel my role is to direct patients rather than discuss risk information about treatments | |  | |  | |  | |  | |  | |  |
| 1. I feel “competent” in involving patients in decision making | |  | |  | |  | |  | |  | |  |
| 1. I feel confident in discussing risk information about treatments with patients | |  | |  | |  | |  | |  | |  |
| 1. I have found that patients respond positively to involvement in decision making | |  | |  | |  | |  | |  | |  |
| 1. Lack of time is a major problem in discussing treatment decisions with patients | |  | |  | |  | |  | |  | |  |
| 1. Lack of available data is a major problem in trying to share decisions | |  | |  | |  | |  | |  | |  |
| 1. Many of my patients expect specific information to be provided in discussions about treatments | |  | |  | |  | |  | |  | |  |

# Any other comments

## Please write in the box below anything you would like to add about guidelines for referral of adults to a surgeon for non-urgent conditions

# About you

## Are you:

| Male |  | 1 |
| --- | --- | --- |
| Female |  | 2 |

## To which of the following age groups do you belong?

| 25 - 34 |  | 1 |
| --- | --- | --- |
| 35 - 44 |  | 2 |
| 45 - 54 |  | 3 |
| 55 - 64 |  | 4 |
| 65 or over |  | 5 |

## How many years is it since you qualified?

| 0 - 9 |  | 1 |
| --- | --- | --- |
| 10 - 19 |  | 2 |
| 20 – 29 |  | 3 |
| 30 or more |  | 4 |
|  |  |  |

## Including yourself, how many fully qualified GPs are there in your practice?

Please include part-time and salaried GPs.

| 1 |  | 1 |
| --- | --- | --- |
| 2 - 3 |  | 2 |
| 4 - 5 |  | 3 |
| 6 - 7 |  | 4 |
| More than 7 |  | 5 |

## What is the size of your personal list?

| Less than 1000 |  | 1 |
| --- | --- | --- |
| 1000 -1999 |  | 2 |
| 2000 - 2999 |  | 3 |
| 3000 or more |  | 4 |
| No personal list |  | 5 |

## Are you a member of any of the following professional organisations?

| RCGP |  | 1 |
| --- | --- | --- |
| BMA |  | 2 |
| Other professional society/societies |  | 3 |
| *(Please state which)* |  |  |

**Thank you very much for your time**

**Please return to:**

**FREEPOST**

**Centre for Health Sciences**

**2 Newark Street**

**Whitechapel**

**London**

**E1 2AT**
